# Feasibility of a SOFA-Derived Organ Dysfunction Score in Cats with Systemic Inflammatory Response Syndrome

**DOI:** 10.3390/life16091575

**Published:** 2026-09-21

**Authors:** Andrei-Răzvan Codea, Alexandra Biriș, Mihaela Niculae, Alina-Diana Hașaș, Daniela Neagu, Cristian Popovici, Mircea Mircean

**Affiliations:** Department of Clinical Sciences, Faculty of Veterinary Medicine, University of Agricultural Sciences and Veterinary Medicine Cluj-Napoca, Calea Mănăștur 3–5, 400372 Cluj-Napoca, Romania; alexandra.biris@usamvcluj.ro (A.B.); alina.hasas@usamvcluj.ro (A.-D.H.); daniela.neagu@usamvcluj.ro (D.N.); cristian.popovici@usamvcluj.ro (C.P.); mircea.mircean@usamvcluj.ro (M.M.)

**Keywords:** cat, systemic inflammatory response syndrome, organ dysfunction score, cross-species adaptation, threshold calibration, pulse oximetry, oxyhaemoglobin dissociation curve, emergency and critical care

## Abstract

The Sequential Organ Failure Assessment (SOFA) score grades dysfunction in six components in people, using thresholds fixed against human reference ranges and a human case mix. Whether those numbers transfer to cats has not been examined. Of 52 cats assessed for eligibility, 23 met predefined systemic inflammatory response syndrome criteria, 22 of them non-septic, and an adapted score was calculated at admission from non-invasive measurements and one venous sample. A complete score was obtained in every cat. Under the applied bilirubin thresholds, the hepatic sub-score exceeded zero in 21 of 23 cats and, with the renal component, accounted for 63.0% of the mean total score; removing that component lowered the median total from 6.0 to 3.0 and left three cats scoring zero. Under stated physiological assumptions, translating the original PaO_2_/FiO_2_ thresholds through the feline oxyhaemoglobin dissociation curve placed the first respiratory threshold near 91%, rather than the 95% applied here, and removed the respiratory point from six cats. Five of the 23 cats did not survive to discharge, too few for any outcome analysis, and none was attempted. The framework was measurable in this setting, but its thresholds describe admission abnormalities rather than organ dysfunction and require feline-specific calibration.

## 1. Introduction

Systemic inflammatory response syndrome (SIRS) describes a generalised inflammatory reaction of the host that may follow infectious or non-infectious insults and that can be accompanied by dysfunction of several organ systems [1,2]. The concept was defined in human medicine and transferred to small animal practice, where species-specific clinical and haematological criteria have been proposed [3]. Cats are considered more difficult to identify than dogs because the presentation is frequently hypodynamic, with bradycardia and hypothermia rather than tachycardia and fever, and because the accompanying signs are otherwise non-specific [4,5].

Because SIRS criteria alone convey limited information about severity, scoring systems have been developed to grade illness in critically ill animals. In cats, the Feline Acute Patient Physiologic and Laboratory Evaluation (APPLE) score stratifies mortality risk from a set of weighted variables [6], and a count of dysfunctional organ systems has been examined in feline sepsis [7]. Neither instrument was designed primarily to grade the severity of dysfunction within individual organ systems or to describe the distribution of organ system involvement.

The Sequential Organ Failure Assessment (SOFA) score was designed for that purpose in people. It assigns a value between 0 and 4 to each of six components, the respiratory, cardiovascular, hepatic, renal, coagulation and neurological components, and the sum describes the overall recorded burden [8]. Each component is graded from a single measured variable, so these labels denote the variable scored rather than a complete assessment of the corresponding organ system. The score is associated with mortality in human intensive care [9,10] and forms part of the current consensus definition of sepsis [11]. Its parameters and thresholds were presented by the original authors as provisional and open to revision [8], and an update has since been proposed [12]. The thresholds attached to those variables were placed against human reference ranges and against the case mix of human intensive care.

Adapting such an instrument to another species raises a question that is separate from whether the instrument is useful. The architecture of the score, six components, each graded from 0 to 4 and summed, carries across species without difficulty. The numbers attached to that architecture do not: every threshold was placed against a human distribution of the measured variable, and the same value means something different in a species with different reference intervals and a different haemoglobin oxygen affinity. This article is concerned with that second question, the calibration of the thresholds rather than the performance of the score. Whether a SOFA-derived framework offers clinical value in cats beyond the instruments already available for this species, such as the feline APPLE score [6], is a further question that the present data do not address.

In veterinary medicine, the SOFA score has been applied to dogs. Ripanti and colleagues modified the bilirubin and creatinine thresholds and reported that the score differed between survivors and non-survivors among critically ill dogs, with serial assessment over the first three days adding information [13]. Kalogianni and colleagues calculated the score at admission and daily over the first four days of hospitalisation in 35 dogs with parvoviral enteritis, of which nine did not survive; the score rose during hospitalisation in non-survivors and fell in survivors, and the admission value separated the outcome groups with an area under the curve of 0.797 [14]. A separate canine study did not calculate the score itself but examined new-onset organ dysfunction in dogs with systemic inflammation [15]. To our knowledge, no prospective evaluation of the feasibility and component behaviour of a SOFA-derived score has been reported in cats, and the adaptations used in the canine studies differ from one another. Neither canine study examined whether the thresholds it applied correspond to the physiological quantities of the original instrument. The term SOFA-derived is used throughout this article for a score that retains the architecture of the original instrument, six components each graded from 0 to 4 and summed, but replaces two variables that cannot be obtained without arterial sampling and re-specifies the thresholds of the remaining components. It is not the original score, and its grades are not interchangeable with those of the human instrument.

Two obstacles recur in any veterinary adaptation. The respiratory component of the original score requires the ratio of arterial oxygen tension to inspired oxygen fraction, which is rarely obtainable in an emergency setting, and the cardiovascular component requires mean arterial pressure, whereas Doppler sphygmomanometry, identified in current consensus guidance as an appropriate indirect method for conscious cats [16], returns systolic pressure only. Peripheral oxygen saturation and systolic arterial pressure have been substituted, but the substituted thresholds have not been examined against the physiological quantities they replace. This is of particular interest in cats because feline haemoglobin has a lower oxygen affinity than canine or human haemoglobin, so that a given oxygen tension corresponds to a lower saturation [17].

This study was therefore designed as a small, single-centre, descriptive study of threshold behaviour rather than as an evaluation of prognostic performance. The first objective was to determine whether a SOFA-derived organ dysfunction score could be calculated at admission in cats meeting predefined SIRS criteria using only non-invasive measurements and a single venous sample. The second was to describe how the applied thresholds behaved in that cohort, in particular, how the recorded burden was distributed between components and how far the total score depended on any one of them. The third was to compare the applied respiratory thresholds with a theoretical SpO_2_-based translation of the original PaO_2_/FiO_2_ thresholds under stated physiological assumptions. No prognostic objective was pursued, and no analysis of the association between the score and the outcome was undertaken.

## 2. Materials and Methods

### 2.1. Study Design and Setting

This was a prospective, observational, single-centre cohort study conducted at the Emergency Hospital of the Faculty of Veterinary Medicine, University of Agricultural Sciences and Veterinary Medicine Cluj-Napoca, Romania, between November 2025 and April 2026. Twenty-one animals were client-owned, and two were presented as strays with no known owner. All animals were housed and treated according to the clinical needs of the individual patient, with analgesia and supportive care prescribed by the attending clinician independently of the study. No procedure was performed for the purposes of the study alone; every measurement formed part of the standard diagnostic evaluation of a critically ill patient.

The protocol and the inclusion criteria were fixed before enrolment, and cases were enrolled consecutively as they presented. The sample was one of convenience. No a priori sample size calculation was performed, and the number of animals studied was determined by the number of eligible presentations during the enrolment period rather than by a target precision.

Serial score assessment was initially planned; however, repeated measurements were incomplete under routine emergency conditions. The primary analysis was therefore restricted to the admission score.

### 2.2. Study Population and Inclusion Criteria

Cats admitted to the intensive care unit were eligible if they met at least two of the four species-specific SIRS criteria: a rectal temperature of 39.7 °C or above or below 37.8 °C; a heart rate of 225 beats per minute or above or 140 beats per minute or below; a respiratory rate of 40 breaths per minute or above; and a leukocyte count of 19.5 × 10^9^/L or above or 5 × 10^9^/L or below, or band neutrophils of 5% or more [4]. These criteria were originally proposed with a requirement of three of four, whereas later work has applied a threshold of two [5]. Two criteria were required here in order not to exclude hypodynamic presentations in which bradycardia and hypothermia may coexist with an otherwise unremarkable leukogram. This modification was prespecified and was intended to broaden inclusion rather than to reproduce the original three-of-four SIRS definition. The consequences of this choice are addressed in the limitations.

Band neutrophils were not enumerated because a manual differential count was not performed on the study samples. The leukocyte criterion was therefore assessed on the total leukocyte count alone, and no band count, relative or absolute, contributed to eligibility. That criterion was met by 11 of the 23 cats included, and in only one animal was it required in order to reach the two-criterion threshold.

The resulting population is a heterogeneous emergency cohort defined by the two-of-four criteria set out above rather than a feline sepsis cohort. Meeting these criteria describes a systemic inflammatory response and does not indicate infection, so the cats included were not septic by virtue of enrolment; one of the 23 animals (4.3%) was classified as septic. Classification as septic or non-septic rested on the suspected underlying condition and the clinical assessment since microbiological confirmation was not obtained systematically. Cats assessed for eligibility were not enrolled if they met fewer than two of the four criteria and were excluded if any variable required for the score was unavailable; the number assessed and the number not enrolled on each ground are reported in Section 3.1. Repeat admissions were treated as separate cases only when they corresponded to distinct episodes with a different underlying condition.

### 2.3. Data Collection and Clinical Measurements

Signalment, presenting complaint, primary diagnosis, clinical findings at admission, length of hospitalisation and the outcome were recorded from the clinical records. Peripheral oxygen saturation was measured by pulse oximetry with a clip sensor placed on the ear, connected to a veterinary multiparameter monitor (LifeVet 8C, Eickemeyer KG, Tuttlingen, Germany). Systolic arterial pressure was measured at admission by Doppler ultrasonography using a veterinary ultrasonic Doppler unit fitted with an 8 MHz concave probe (EICKEMEYER Ultrasonic Doppler, article 217210, Eickemeyer KG, Tuttlingen, Germany). Measurements were obtained over the left or right dorsal metatarsal artery, with an occlusive cuff positioned proximally to the probe. The cuff width was selected to correspond to approximately 30 to 40% of the limb circumference at the measurement site [16]. Four Doppler readings were obtained for each determination, and their arithmetic mean was used as the recorded systolic arterial pressure. All measurements were performed at admission before fluid therapy and before administration of sedative drugs. No cat received supplemental oxygen at the time of the saturation reading, so an inspired oxygen fraction of 0.21 applies throughout. Pulse oximetry in cats is subject to device-dependent and site-dependent error, and accuracy deteriorates as saturation falls [18]. Venous blood was collected by jugular, cephalic or medial saphenous venepuncture into lithium heparin tubes for biochemistry and into tubes containing ethylenediaminetetraacetic acid for haematology. Total bilirubin and creatinine were measured with a rotor-based veterinary chemistry analyser (element RC, scil animal care company GmbH, Viernheim, Germany) and platelet counts with a five-part differential veterinary haematology analyser (Abacus Vet5, Diatron MI Zrt., Budapest, Hungary), which enumerates platelets by volumetric impedance. Automated platelet counts were not confirmed by blood smear examination, and no smear was reviewed for platelet aggregates. In a proportion of cases, bilirubin and creatinine were obtained instead from a critical care analyser (Stat Profile pHOx Plus, Nova Biomedical, Waltham, MA, USA). No cat received medication expected to alter consciousness before neurological assessment, and the modified Glasgow Coma Scale (mGCS) was assessed without sedation [19].

The mGCS was developed and evaluated in dogs with head trauma [19]; the citation identifies the instrument and is not offered as evidence of validity in this population. The mGCS was included as an exploratory neurological component because it provided a clinically feasible standardised neurological assessment, and its inclusion should not be interpreted as implying validation of the instrument in cats with systemic inflammation.

### 2.4. The Applied Organ Dysfunction Score

The original score requires the ratio of arterial oxygen tension to inspired oxygen fraction for the respiratory component and mean arterial pressure for the cardiovascular component [8]. Neither was obtainable in this setting, and pragmatic surrogates were substituted. The scale applied, together with the basis on which each threshold was selected, is given in Table 1. Three origins are distinguished there. A threshold is described as adapted where it was carried over unchanged from the original score, as modified where a published feline reference framework was used to reset it, and as empirically selected where it was placed by the investigators against the reference intervals of the study laboratory or against the measurement range of the method, without external validation. The thresholds are pragmatic working values and are not validated cut-offs for cats; a sub-score above zero under this scale should not be read as a validated diagnosis of organ dysfunction.

The cardiovascular component was not a direct adaptation of the original SOFA variable. Because mean arterial pressure (MAP) was unavailable, systolic arterial pressure (SAP) measured by Doppler sphygmomanometry was used as a pragmatic surrogate, supplemented by vasopressor treatment. Accordingly, the cardiovascular sub-score should not be interpreted as equivalent to the original MAP-based SOFA cardiovascular component. Indirect systolic pressure was interpreted against published Doppler reference values for conscious cats [20]. Doppler sphygmomanometry was used exclusively, so the cohort is internally consistent in this respect, although indirect measurement in conscious cats remains imperfect and device-dependent [21]. The assignment rule was as follows: a cat with a SAP of 105 mmHg or above and no vasopressor support was scored zero; in cats not receiving vasopressors, any SAP below 105 mmHg was assigned one cardiovascular point, irrespective of the absolute pressure value; grades 2 to 4 were determined solely by the vasopressor agent and dose given in Table 1, and where both criteria applied the higher grade was assigned.

**Table 1:** Organ dysfunction score applied in this study, with the basis for each threshold.

Component	Parameter	0	1	2	3	4	Basis for Threshold Selection
Respiratory	SpO_2_ (%)	96 to 100	91 to 95	86 to 90	75 to 85	below 75	Empirically selected; pragmatic SpO_2_ substitution for the original PaO_2_/FiO_2_ variable; physiological consistency examined in this study
Cardiovascular	SAP (mmHg) or vasopressor	SAP ≥ 105 mmHg and no vasopressor support	SAP < 105 mmHg	dopamine 5 or less, or any dobutamine	dopamine above 5, or epinephrine or norepinephrine 0.1 or less	dopamine above 15, or epinephrine or norepinephrine above 0.1	Systolic threshold empirically selected; vasopressor grades adapted unchanged from the original score; indirect SAP is not equivalent to the original MAP-based component
Hepatic	Total bilirubin (mg/dL)	below 0.2	0.2 to 0.5	0.6 to 1.0	1.1 to 2.0	above 2.0	Empirically selected from the feline laboratory reference interval; not adapted from the original human cut-offs
Renal	Creatinine (mg/dL)	below 1.6	1.6 to 2.1	2.2 to 3.6	3.7 to 5.0	above 5.0	Modified from the IRIS staging system for cats [22]; baseline renal status often unavailable
Coagulation	Platelets (10^9^/L)	above 150	150 or below	100 or below	50 or below	20 or below	Empirically selected from the study laboratory reference interval
Neurological	mGCS	18	15 to 17	11 to 14	6 to 10	below 6	Empirically selected grouping of mGCS values; mGCS replaces the neurological variable of the original score

SpO_2_, peripheral oxygen saturation; SAP, systolic arterial pressure; PaO_2_, arterial oxygen tension; FiO_2_, fraction of inspired oxygen; MAP, mean arterial pressure; mGCS, modified Glasgow Coma Scale; IRIS, International Renal Interest Society. Vasopressor doses are in µg/kg/min and refer to administration for at least one hour. When both systolic arterial pressure and vasopressor support were available, the higher cardiovascular grade was assigned. None of these thresholds has been validated against the outcome in cats. Each component is graded from a single measured variable, and the component names follow the convention of the original score; they do not denote a comprehensive assessment of the corresponding organ system. The coagulation component in particular reflects the platelet count alone rather than a haemostatic profile, and the hepatic and renal components reflect total bilirubin and creatinine, respectively.

All thresholds were prespecified for this study and should be considered working thresholds rather than validated feline definitions of organ dysfunction.

Renal categories were informed by the staging system of the International Renal Interest Society [22]. Coagulation categories were set against the reference intervals in use in the study laboratory for the analytical platforms described above. Because no feline-validated SOFA hepatic thresholds were available, bilirubin categories were defined a priori as exploratory working thresholds informed by the laboratory reference interval. These categories were not intended to represent validated definitions of hepatic organ dysfunction. The laboratory reference interval for total bilirubin was 0.1 to 0.4 mg/dL, so that the first applied hepatic grade begins at a concentration that lies within that interval.

### 2.5. Theoretical Translation of the Original Respiratory Thresholds

Substituting saturation for oxygen tension changes the quantity measured, so the applied respiratory thresholds were compared with a theoretical translation of the thresholds they replace. The oxygen tension corresponding to each original cut-off was obtained at a fraction of the inspired oxygen of 0.21 and converted to the corresponding feline saturation with the Hill equation, using a P50 of 35.6 mmHg determined tonometrically for feline haemoglobin [17] and a Hill coefficient of 2.7. The translation assumes standard pH, carbon dioxide tension and temperature. All cats were breathing room air at the time of measurement, so an inspired oxygen fraction of 0.21 applies. Because no feline-specific Hill coefficient is available, the translation was repeated across a plausible range of 2.5 to 3.0, and the resulting interval is reported alongside the point value. It is a theoretical physiological comparison under stated assumptions and is not an outcome-validated feline respiratory scale. Establishing whether either set of cut-offs identifies respiratory dysfunction in cats would require paired arterial blood gas and pulse oximetry measurements across the relevant saturation range, which were not obtained in this study. The resulting values are given in Table 2.

### 2.6. Outcome

The outcome was survival to hospital discharge. Cats that died spontaneously and cats euthanised on veterinary advice during hospitalisation were classified together as non-survivors. One of the five non-survivors was euthanised, and that animal is identified separately wherever the outcome groups are described.

### 2.7. Statistical Analysis

Continuous variables are reported as the median and interquartile range, and categorical variables as counts and percentages. Because the cohort comprises 23 animals and five non-survival events, the analysis is descriptive throughout. No hypothesis test was performed, no cut-off was derived, and no *p*-value, confidence interval or effect estimate is reported anywhere in this article. Where values are given separately for survivors and non-survivors, they describe the cohort, and no comparison between those groups is made or implied. For descriptive completeness, the sensitivity and specificity of the applied total score at each attainable cut-off are reported, without inferential testing, in Table S1.

Two laboratory values were censored at the reporting limits of the analytical platform and were handled as such: the systolic pressure of one cat was recorded only as above 105 mmHg and the creatinine of one cat only as above 12 mg/dL. Both were sufficient to assign the corresponding sub-score unambiguously, and neither was used as an exact value in any descriptive summary; no mean or median creatinine concentration is reported for that reason.

Two recalculations of the total score are reported, both descriptive. The influence of the hepatic component was examined by recalculating every total after removing that component, and the influence of the respiratory thresholds by recalculating every total under the theoretically translated thresholds of Section 2.5. Neither recalculation was evaluated against the outcome.

Data were compiled in Microsoft Excel for Microsoft 365, version 16.0 (Microsoft Corporation, Redmond, WA, USA). All analyses were performed in Python version 3.12.3 with NumPy 2.4.4, SciPy 1.17.1 and pandas 3.0.2.

## 3. Results

### 3.1. Study Population

Fifty-two cats were assessed for eligibility during the study period. Twenty-nine met fewer than two SIRS criteria and were not enrolled, and no cat was excluded because a variable required for the score was unavailable, so 23 cats were included. Eighteen (78.3%) survived to discharge and five (21.7%) did not. Four of the five died in hospital after cardiopulmonary arrest, on days 2, 4, 11 and 23 after admission, with primary diagnoses of acute kidney injury with suspected ethylene glycol intoxication, polytrauma, gastrointestinal obstruction with suspected aspiration, and concurrent chronic kidney disease, hypertrophic cardiomyopathy and lymphoma, respectively. The fifth, a cat with feline asthma and gastritis, was euthanised on day 3 on the combined grounds of clinical deterioration and the inability of the owner to continue funding treatment. Necropsy was not performed in any of the five animals, so the terminal event could not be attributed to a specific cause beyond the primary diagnosis. The cohort was predominantly non-septic, with 22 of 23 cats (95.7%) classified as non-septic. Characteristics are given in Table 3.

The largest single presenting category was trauma, in seven cats (30.4%), followed by inflammatory and renal conditions in four cats each (17.4%), infectious and idiopathic presentations in two cats each (8.7%), and single cases of toxic, multisystemic, mechanical and septic origin.

The toxic presentation involved acute kidney injury with suspected ethylene glycol intoxication, and the infectious presentations included one cat with suspected dry-form feline infectious peritonitis and one with feline coronavirus infection and concurrent giardiasis.

### 3.2. Feasibility

A complete score was obtained in all 23 cats. No animal was excluded for a missing variable, and no arterial sample was required. All six components were derived from a physical examination, a pulse oximeter reading, indirect systolic pressure and a single venous sample (Table 1). Every cat met at least two SIRS criteria, with a median of three.

### 3.3. Distribution of the Admission Score

**Figure 1:**
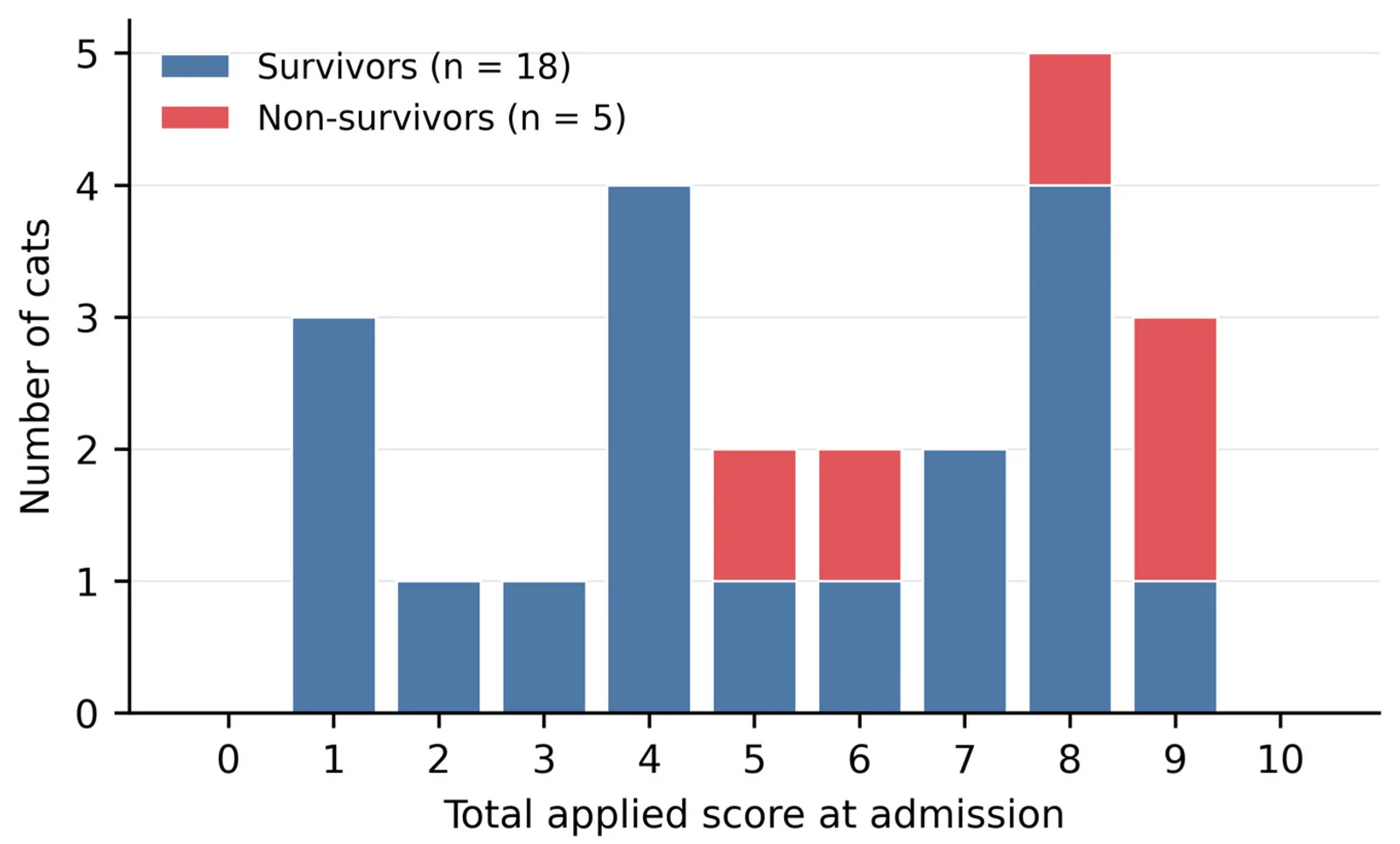
Distribution of the total applied score at admission in 23 cats meeting predefined systemic inflammatory response syndrome criteria, separated by outcome. Each bar represents the number of cats with the corresponding total score. This descriptive distribution should not be interpreted as establishing a prognostic cut-off.

The total score ranged from 1 to 9, with a median of 6.0 and an interquartile range of 4.0 to 8.0 (mean 5.52). The distribution by outcome is shown in Figure 1.

### 3.4. Component Profile

**Figure 2:**
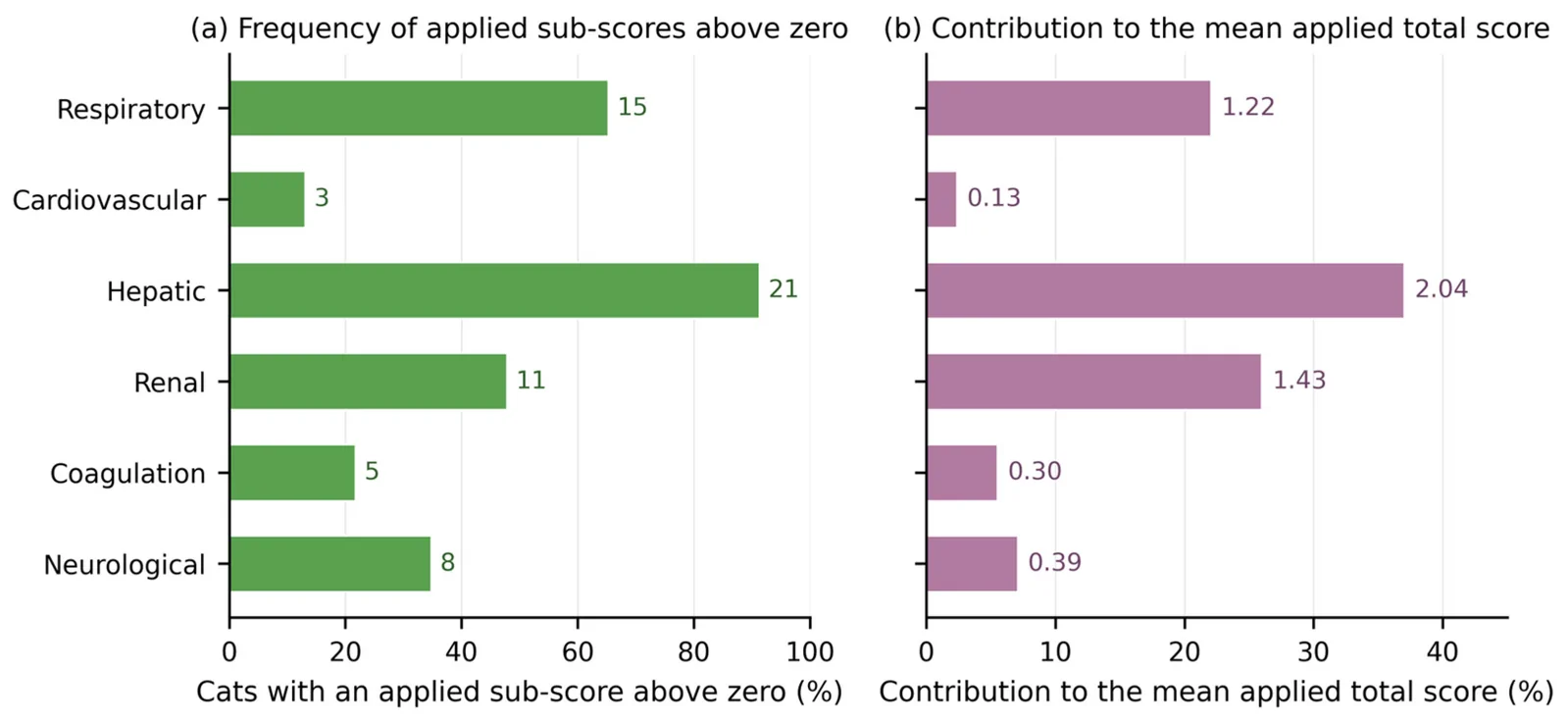
Frequency and contribution of each component under the thresholds applied in this study. (**a**) Proportion of cats with an applied sub-score above zero, with counts beside each bar. (**b**) Percentage of the mean applied total score contributed by each component, with the mean sub-score beside each bar. A sub-score above zero should not be interpreted as a validated diagnosis of organ dysfunction.

The hepatic sub-score was above zero in 21 of 23 cats under the applied bilirubin thresholds and contributed more to the mean total score than any other component. The renal sub-score was second by contribution. Cardiovascular and coagulation sub-scores were above zero in few animals. No cat received vasopressor support, so cardiovascular grades 2 to 4 were not attainable in this cohort, and the three cardiovascular sub-scores above zero were assigned on the basis of systolic arterial pressure alone. Component frequencies, contributions and distributions by outcome are given in Table 4 and shown in Figure 2.

Of the 21 of 23 cats with a hepatic sub-score above zero, five had a bilirubin concentration above 2.0 mg/dL, the value defining the highest applied grade. Total bilirubin ranged from 0.0 to 8.8 mg/dL, with a median of 0.7 mg/dL. Only two cats had a concentration below 0.5 mg/dL, so that raising the first applied grade from 0.2 mg/dL to the upper laboratory reference limit of 0.4 mg/dL, or to 0.5 mg/dL, would leave the same 21 of 23 cats with a sub-score above zero; 15 of the 23 cats had a concentration of 0.6 mg/dL or above, and six of the 23 had a concentration of 1.0 mg/dL or above. Eleven of the 23 cats had a creatinine concentration at or above 1.6 mg/dL, the value defining the first applied renal grade; kidney disease formed part of the primary diagnosis in seven cats, and chronic kidney disease was documented in one. The hepatic and renal components together accounted for 63.0% of the mean applied total score.

Removing the hepatic component from the total reduced the median score from 6.0 (interquartile range 4.0 to 8.0) to 3.0 (2.0 to 6.0) and the mean from 5.52 to 3.48 points. The total changed in the 21 cats with a hepatic sub-score above zero, and three cats were left with a total of zero, their entire recorded burden having come from that component alone. Under the reduced total, the renal component became the largest contributor, accounting for 33 of the 80 points remaining across the cohort, followed by the respiratory component with 28.

### 3.5. Comparison of Respiratory Thresholds

**Figure 3:**
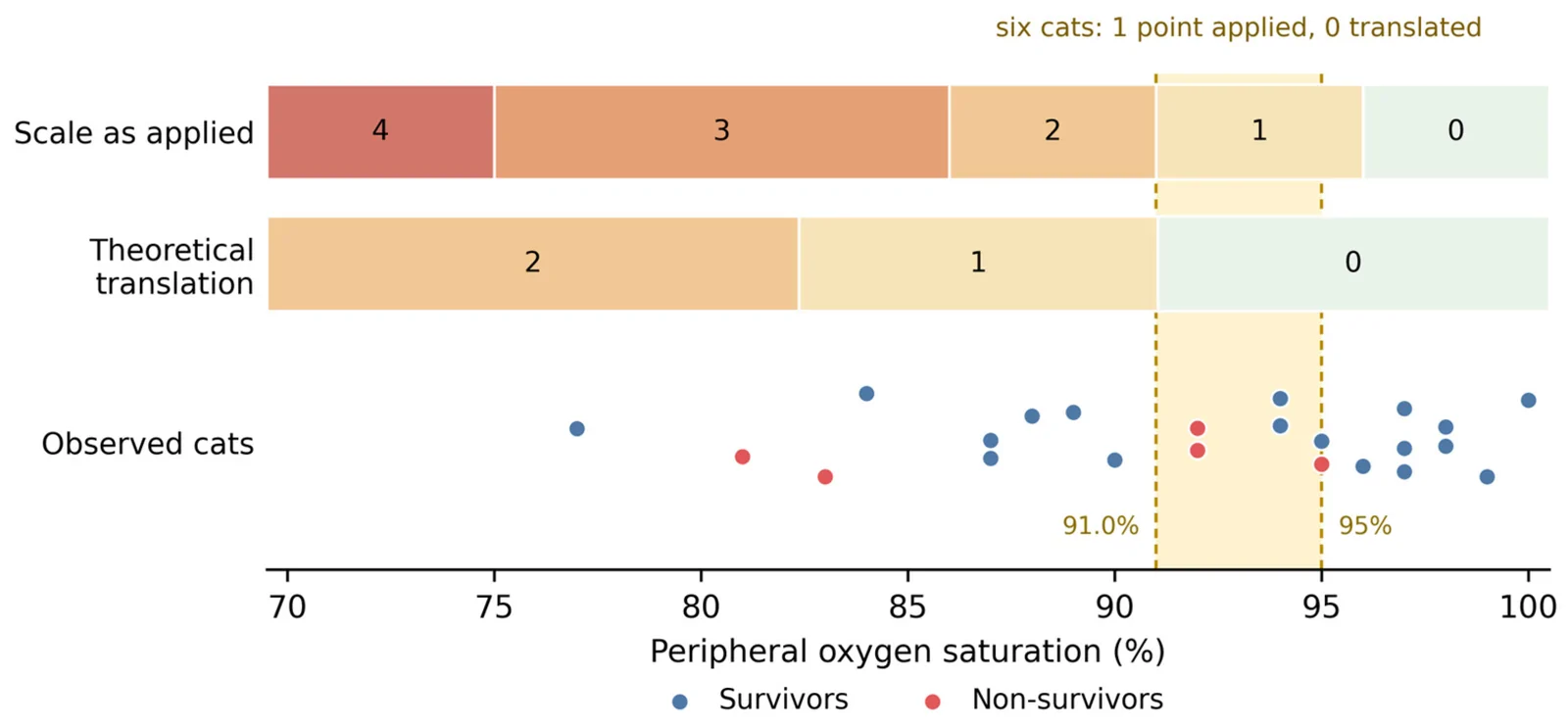
Physiological comparison of respiratory thresholds. Each point is one cat, positioned by its admission peripheral oxygen saturation and coloured by the outcome. The upper band shows the scale applied in this study. The lower band represents a theoretical SpO_2_-based translation of the original PaO_2_/FiO_2_ cut-offs, derived using the Hill equation under the assumptions stated in the Section 2; it is not an outcome-validated feline respiratory scoring scale. Translated grades 3 and 4 fall below the plotted range.

A respiratory sub-score above zero was recorded in 15 of 23 cats under the applied scale, contributing 22.0% of the mean total score. Six of these cats were assigned their point at saturations of 92%, 92%, 94%, 94%, 95% and 95%. Under the theoretical translation in Table 2, all six would have scored zero, since the translated first grade begins at 91.0% or below. Figure 3 shows the observed saturations against both scales.

Applying the theoretically translated thresholds reduced the proportion of cats with a respiratory sub-score above zero from 15 of 23 to 9 of 23, reduced the mean respiratory sub-score from 1.22 to 0.48 points and reduced the mean total score from 5.52 to 4.78 points. The total score changed in 15 of the 23 cats by a median of one point; these are not the same 15 animals as those with a respiratory sub-score above zero. Across Hill coefficients of 2.5 to 3.0, the translated first grade lies between 89.5% and 92.9%, so that between four and all six of these cats would score zero. Under the stated assumptions, the most severe translated category lies outside the range in which pulse oximetry is expected to provide reliable clinical measurements in living cats; the practical measurement range of the remaining translated categories requires empirical assessment.

### 3.6. Admission Score by Outcome

Five of the 23 cats did not survive to hospital discharge. Their admission scores were higher, with a median of 8.0 (interquartile range 6.0 to 9.0) against 4.5 (3.2 to 7.8) in the 18 survivors. Excluding the cat that was euthanised, the median among the remaining four non-survivors was 7.5 (5.8 to 9.0). With five events, these medians describe the cohort and nothing further; no comparison between the groups was performed, and no association between the score and outcome is claimed.

### 3.7. Secondary Descriptive Observations

The following observations are descriptive. Among the 21 cats whose age was recorded, admission scores tended to be higher in older animals: the median was 4.5 in the 10 cats under three years of age and 8.0 in the 11 cats of three years or more. Scores showed no apparent relation to the length of hospitalisation, and the length of stay was similar in the two outcome groups, with medians of 5 days in survivors and 4 days in non-survivors. Median scores across the five diagnostic categories represented by more than one animal ranged from 4.0 to 7.0, in subgroups of two to seven animals.

## 4. Discussion

This study evaluated whether a SOFA-derived organ dysfunction score can be applied at admission to cats meeting predefined SIRS criteria and examined how the applied thresholds behave in this population. Three observations follow. The score was calculable in every animal using equipment already present in an emergency hospital. The recorded burden fell predominantly on the hepatic and renal components. And, under the stated physiological assumptions, the applied respiratory thresholds do not reproduce the original PaO_2_/FiO_2_ categories when translated to feline SpO_2_. Taken together, these observations concern the calibration of the instrument rather than its performance.

### 4.1. Component Profile and Threshold Behaviour

The behaviour of the hepatic component illustrates a general difficulty in transferring a scoring system between species. In the original instrument, the first hepatic grade begins at a bilirubin concentration of 1.2 mg/dL [8], a value fixed against human reference ranges and a human intensive care case mix. The categories applied here began several times lower, within the feline laboratory reference interval, and a sub-score above zero followed in almost every animal. Raising the first grade to the upper reference limit would not have changed that because the distribution of measured concentrations was itself shifted; so, the frequency is a property of the cohort rather than an artefact of where a single cut-off was placed. A component positive in almost every patient cannot discriminate on positive or negative status alone, whatever its physiological rationale, although variation in severity within the component may still carry information that a cohort of this size cannot reveal. The wider point is that a score inherits the epidemiology of the population in which its thresholds were fixed. Transferring the architecture of an organ dysfunction score is straightforward; transferring the numbers attached to it is not.

The size of that dependence can be stated directly. Removing the hepatic component reduced the median total from 6.0 to 3.0, and three cats were left with no recorded burden at all; so, in those animals, the whole score had been generated by a bilirubin concentration lying at or close to the laboratory reference interval. A total assembled in this way records that a measured variable crossed a prespecified line. It does not establish that the organ was dysfunctional, and the two should not be equated when the line itself was drawn against another species.

The same reservation applies to the renal component. Increased creatinine at a single time point does not establish acute kidney injury attributable to the current episode, and kidney disease formed part of the primary diagnosis in almost a third of the cohort, with chronic kidney disease documented in one animal. Hepatic and renal dysfunction have been reported in cats [7] and dogs [23] with sepsis and, in those populations, have been associated with the development of multiorgan dysfunction. The present cohort, however, was predominantly non-septic and was not designed to evaluate sepsis-associated organ dysfunction; therefore, the hepatic and renal abnormalities observed here should not be interpreted as evidence of sepsis-related organ dysfunction.

A further point applies to both components. Bilirubin and creatinine were measured once, at admission, and no pre-admission values were available, so an increased concentration cannot be attributed to the current inflammatory episode rather than to a pre-existing condition. Kidney disease already formed part of the primary diagnosis in seven of the 23 cats, a proportion consistent with the baseline prevalence expected in a feline emergency population, so the renal sub-scores recorded here are as compatible with pre-existing renal disease as with acute injury. What the applied score describes is the burden of laboratory and physiological abnormality present at admission, not new-onset organ dysfunction.

The practical corollary is that a bilirubin and a creatinine concentration, obtainable from a single venous sample, drive most of the applied score in this population. Whether this distribution is clinically appropriate depends on whether the applied thresholds are valid for feline emergency populations, which has not yet been established.

### 4.2. The Applied Respiratory Thresholds

Substituting peripheral saturation for the ratio of arterial oxygen tension to inspired fraction is a reasonable practical decision and is the only way the component can be scored without an arterial sample. The question is what thresholds should attach to the substitute. Feline haemoglobin has a P50 of about 35.6 mmHg, higher than the canine or human value, so its dissociation curve is displaced to the right, and a given oxygen tension corresponds to a lower saturation [17]. Translating the original cut-offs through that curve, under the assumptions stated in the Section 2, places every grade at a lower saturation than the value applied here, and the displacement widens with severity (Table 2).

The consequence is visible in this cohort. Six cats received a respiratory point at saturations between 92% and 95%, values that, in isolation, may not indicate clinically overt respiratory dysfunction and that the translated thresholds score as zero. Applying the translated thresholds removed rather more than a third of the recorded respiratory positives and about three quarters of a point from the mean total score. A component contributing a fifth of the score merits closer attention to where its first grade begins. The problem is not peculiar to cats. The same substitution was proposed in human intensive care for the same practical reasons, and it had to be derived rather than assumed: saturation-based ratios corresponding to the arterial cut-offs for acute lung injury and acute respiratory distress syndrome were established empirically from paired measurements, and the resulting values are not numerically interchangeable with the arterial ones [24]. If a substitution made within a single species required its own derivation, a substitution that also crosses a species boundary, and with it a difference in haemoglobin oxygen affinity, cannot reasonably be made by carrying the original numbers across unchanged.

Two qualifications are necessary. First, the translation is theoretical: it assumes room air; standard pH, carbon dioxide tension and temperature; and a single Hill coefficient, none of which held uniformly in a cohort that included hypothermic and probably acidotic animals, and it has not been validated against the outcome. It establishes the direction and approximate magnitude of a discrepancy, not a corrected scale. Second, no attempt was made to establish which scale performs better against the outcome because a cohort with five events cannot support that comparison. The argument advanced here concerns what the component measures rather than how it performs, and the two should not be conflated.

A further practical point is that the translated saturation for the most severe original grade lies far below the range in which pulse oximetry is expected to give reliable readings in living cats, and pulse oximetry is in any case least accurate at low saturations and during hypoperfusion, with accuracy varying markedly between devices and probe sites in cats [18]. These findings support independent feline calibration of the respiratory component rather than direct transfer of the original SOFA thresholds. That calibration will require paired arterial blood gas and pulse oximetry measurements in cats. Until such data exist, the saturation cut-offs applied here, and the translated values set against them, are working assumptions rather than established indicators of respiratory dysfunction in this species.

### 4.3. What These Data Do Not Address

Admission scores were higher among cats that did not survive to discharge. With five events, a cohort of this size cannot establish whether that difference reflects an association, and no analysis capable of addressing the question was attempted. The appropriate conclusion is that the study does not address prognosis, not that the score lacks prognostic value. Single-centre cohorts of this size are common in veterinary emergency research, and the risk they carry is not that they fail to reach a significance threshold but that an isolated favourable estimate is carried forward as though it had been validated. For that reason, no measure of discrimination and no candidate cut-off are reported.

Whether a graded organ dysfunction score adds anything to the instruments already available for cats is a related question that these data also leave open. The feline APPLE score was developed and evaluated to stratify mortality risk in this species [6], and a count of dysfunctional organ systems has been examined in feline sepsis [7]. Neither was calculated alongside the applied score in the present cohort, so no statement about incremental value can be made, and none is made. A study designed for that comparison, applying both instruments to the same animals, would be required.

### 4.4. Comparison with the Canine Literature

The canine studies of the score used different adaptations from one another and from the scale applied here, and both examined serial measurements rather than a single admission value [13,14]. Differences between their findings and the present observations should therefore be attributed to species, case mix, study design and scoring adaptation together, rather than attributed to any single factor. The translation presented in Table 2 could be repeated for dogs using a canine P50 and would be expected to give a smaller displacement since canine oxygen affinity is closer to the human value. Comparability between veterinary applications of the score is unlikely to improve until such a check becomes routine. Read alongside the human experience, in which the thresholds of the original score were presented as provisional [8] and have since been reconsidered [12], the pattern is consistent. An organ dysfunction score is not a fixed instrument but a set of thresholds that has to be maintained against the population it is used in. Veterinary adaptations have so far imported the architecture and the numbers together, and the numbers are the part that does not travel.

### 4.5. Clinical Implications

The score was obtainable in every animal without arterial puncture and using equipment already present in a hospital equipped for intensive care. On this basis, the framework is feasible as a structured description of measured organ system variables at admission. Feasibility of measurement, however, should not be equated with validity of the transferred thresholds, as illustrated by the hepatic and respiratory findings. Nor should a sub-score above zero be read as organ dysfunction. What the framework records is that a measured variable fell outside a prespecified range; whether that abnormality corresponds to clinically relevant dysfunction of the organ concerned is a separate claim and one this study does not test. The framework may support structured clinical documentation and communication, although these potential uses were not evaluated in this study. A graded score would complement rather than replace the instruments already available for cats by describing where the recorded burden falls and how severe it is within each component, but only once its thresholds have been derived for this species. What these data do not support is use of the total score to inform a decision about an individual cat, and no such use is proposed.

### 4.6. Limitations

The cohort is small, with five outcome events, and comes from a single centre, so all outcome-related estimates are imprecise, and the case mix reflects a university emergency service. The population is heterogeneous and predominantly non-septic, with trauma the largest single category, and only one cat classified as septic, and it is therefore not representative of feline sepsis. Requiring two rather than three SIRS criteria was a deliberate choice in favour of sensitivity, and it changed the nature of the cohort rather than merely widening it. A two-criterion entry rule has lower specificity for systemic inflammation than the three-criterion definition from which it derives, and the population it produced is a general emergency and trauma population, in which trauma was the largest single category, rather than the sepsis population the original criteria were framed to identify. The findings reported here should therefore be read as describing the behaviour of a SOFA-derived scoring framework in that population and should not be set directly against studies that applied the three-of-four definition.

The score was calculated once, at admission; serial assessment was planned but could not be completed, and evidence from human medicine [9,12] and from dogs [13,14] suggests that change over time carries information that a single value cannot. An additional limitation is that the admission measurements could not consistently be distinguished from pre-existing abnormalities because pre-admission baseline bilirubin, creatinine and platelet concentrations were not available. Consequently, the applied score describes the burden of threshold-defined physiological and laboratory abnormalities at admission rather than demonstrably new-onset organ dysfunction. Blood pressure was obtained by a single indirect technique in every cat, which removes between-method variation but does not remove the limitations of indirect measurement in conscious cats [21], and the cardiovascular component used systolic rather than mean arterial pressure as a pragmatic surrogate for the original SOFA variable. Treatments administered before hospital admission or before the study measurements were not systematically recorded; consequently, their potential effects on physiological and laboratory variables could not be quantified.

Three measurement limitations affect the variables used. Band neutrophils were not enumerated, so the leukocyte SIRS criterion was applied on the total leukocyte count alone, and the band branch of that criterion was inoperative. Platelet counts were obtained by volumetric impedance and were not confirmed on a blood smear; feline platelets aggregate readily in ethylenediaminetetraacetic acid blood, and in impedance-based analysers, aggregates lower the platelet count while raising the leukocyte count [25]; so, the coagulation sub-score and the leukocyte criterion may both have been affected, in opposite directions, in an unknown number of animals. The hepatic component was represented by total bilirubin alone, as in the original score; alanine aminotransferase, alkaline phosphatase, albumin and bile acids were not part of the prespecified protocol and were not available for the whole cohort, so whether additional hepatic variables would behave differently could not be examined here.

Because euthanasia was included in the non-survivor category, the outcome incorporates both biological survival and decisions taken outside the clinical course; the single euthanasia in this cohort followed both clinical deterioration and a financial constraint on continued treatment, so that outcome cannot be attributed to the disease course alone, which is why that animal is identified separately wherever the outcome groups are described. Necropsy was not performed in any non-survivor, so no terminal event could be attributed to a specific cause beyond the primary diagnosis. The mGCS contains a subjective element. Age was not recorded for the two animals presented as strays, whose age could not be established. The translation of respiratory thresholds was performed under standard physiological assumptions without individual adjustment for temperature, pH or carbon dioxide tension, and used a Hill coefficient that has not been determined for cats, although the conclusion was stable across a plausible range. Pulse oximetry has inherent limitations during hypoperfusion and at low saturations [18].

### 4.7. Future Directions

Three steps follow from these observations. The respiratory and cardiovascular components should be evaluated and, if necessary, recalibrated for cats, with thresholds derived either from species-specific oxygen affinity data or against outcome, and with explicit recognition of the measurement range that pulse oximetry can support. Future studies should prospectively evaluate serial scores at admission, 24 h and 48 h because change in organ dysfunction may carry information that a single admission measurement cannot. The sample size for such work should be derived from a prespecified target for calibration or discrimination rather than from estimates observable in a cohort of the present size, which are too imprecise to plan against. A single centre is in any case unlikely to enrol a sufficient number of cats within a reasonable period, so a multicentre design is the realistic route, using a prespecified, species-calibrated adaptation applied identically at every site and recording baseline organ function where available. Future protocols should also measure additional hepatic variables alongside bilirubin and confirm platelet counts on a blood smear or by an optical method, so that the hepatic and coagulation components can be assessed without the measurement limitations that applied here. Comparison with the feline APPLE score [6] on the same animals would establish whether graded organ dysfunction adds information beyond an instrument already developed for this species.

## 5. Conclusions

In this prospective, single-centre cohort of cats meeting predefined systemic inflammatory response syndrome criteria, a complete SOFA-derived organ dysfunction score could be calculated at admission for every enrolled animal using physical examination findings, pulse oximetry, indirect systolic arterial pressure measurement and a single venous blood sample. This supports the practical feasibility of obtaining the variables required for the applied score within the resources and workflow of a university emergency hospital. Feasibility, however, did not establish the validity of the applied thresholds.

Two features of the applied scale limited the interpretability of its total score. The hepatic sub-score exceeded zero in 21 of 23 cats under the applied bilirubin thresholds and, together with the renal sub-score, accounted for 63.0% of the mean total score; because only two cats had a total bilirubin concentration below the upper laboratory reference limit of 0.4 mg/dL, this frequency reflects the distribution of measured concentrations as well as the position of the first cut-off. Removing that component reduced the median total from 6.0 to 3.0 and left three cats with no recorded burden. For the respiratory component, a theoretical translation of the original PaO_2_/FiO_2_ thresholds through the feline oxyhaemoglobin dissociation curve placed the first threshold at an SpO_2_ of approximately 91.0%, rather than the 95% used in the applied scale, with the higher grades falling at progressively lower saturations, at which pulse oximeter measurements may be unreliable. These values should be regarded as a physiological basis for future testing, not as an outcome-validated feline respiratory scoring scale.

The small, heterogeneous and predominantly non-septic cohort, together with the limited number of non-survival events, precludes any assessment of prognostic performance or derivation of a clinical cut-off, and neither was attempted. The observed component distributions and the theoretical respiratory threshold translation together demonstrate that the applied thresholds cannot yet be considered feline-validated measures of organ dysfunction. Feline-specific threshold calibration, assessment of baseline organ function, serial evaluation during the first 48 h of hospitalisation and multicentre external validation are required before prognostic or clinical use.

## Figures and Tables

**Table 2:** Theoretical translation of the original respiratory thresholds into feline peripheral oxygen saturation, compared with the thresholds applied in this study.

Points	Original Definition	PaO_2_ at FiO_2_ 0.21	Translated Feline SpO_2_	SpO_2_ Threshold Applied Here
1	PaO_2_/FiO_2_ 400 or below	84 mmHg or below	91.0% (89.5 to 92.9)	95% or below
2	PaO_2_/FiO_2_ 300 or below	63 mmHg or below	82.4% (80.6 to 84.7)	90% or below
3	PaO_2_/FiO_2_ 200 or below	42 mmHg or below	61.0% (60.2 to 62.2)	85% or below
4	PaO_2_/FiO_2_ 100 or below	21 mmHg or below	19.4% (17.0 to 21.1)	below 75%

PaO_2_, arterial oxygen tension; FiO_2_, fraction of inspired oxygen; SpO_2_, peripheral oxygen saturation. Translated saturations were obtained with the Hill equation at a P50 of 35.6 mmHg [17] and a Hill coefficient of 2.7, under standard pH, carbon dioxide tension and temperature; values in parentheses are the range obtained across Hill coefficients of 2.5 to 3.0. Under these assumptions, each applied grade is reached at a higher saturation than the original definition, and the difference widens with severity. The translated values are theoretical and have not been validated against the outcome.

**Table 3:** Characteristics of the 23 cats at admission. Values refer to all 23 animals unless a different denominator is given.

Variable	Value
Age, median (IQR), years (*n* = 21)	3.0 (1.5 to 9.0)
Age, range, years (*n* = 21)	0.25 to 14
Age not recorded, both animals presented as strays, n	2
Male, *n* (%)	13 (56.5)
Female, *n* (%)	10 (43.5)
Domestic shorthair, *n* (%)	18 (78.3)
British shorthair, *n* (%)	3 (13.0)
Birman, *n* (%)	2 (8.7)
Body weight, median (IQR), kg	3.7 (3.0 to 4.4)
SIRS criteria met: two, *n* (%)	10 (43.5)
SIRS criteria met: three, *n* (%)	10 (43.5)
SIRS criteria met: four, *n* (%)	3 (13.0)
Classified as septic, *n* (%)	1 (4.3)
Classified as non-septic, *n* (%)	22 (95.7)
Trauma-related presentation, *n* (%)	7 (30.4)
Kidney disease within the primary diagnosis, *n* (%)	7 (30.4)
Lower urinary tract disease within the primary diagnosis, *n* (%)	3 (13.0)
Chronic kidney disease documented, n	1
Retrovirus-positive status recorded, *n* (%)	4 (17.4)
Receiving supplemental oxygen at the saturation reading, n	0
Blood pressure obtained by Doppler at a dorsal metatarsal artery, *n* (%)	23 (100)
Length of stay, median (IQR), days	5 (3.5 to 8)
Length of stay, range, days	2 to 28
Survived to discharge, *n* (%)	18 (78.3)
Did not survive, *n* (%)	5 (21.7)
Euthanised among non-survivors, n	1

IQR, interquartile range; SIRS, systemic inflammatory response syndrome. Age was not recorded for the two cats presented as strays, so every age-related analysis is based on 21 animals. Categories are not mutually exclusive: one cat had both acute kidney injury and lower urinary tract disease. Kidney disease is counted whenever it formed part of the primary diagnosis, irrespective of the aetiological category assigned to the presentation. The count of 7 given here is therefore a different quantity from the 4 cats assigned to the renal aetiological category in the text, and the two are not expected to agree. Systolic arterial pressure was calculated as the mean of four Doppler measurements obtained at admission before fluid therapy and sedation.

**Table 4:** Applied sub-scores by component: frequency, contribution to the mean applied total score and distribution by outcome.

Component	Cats with an Applied Sub-Score > 0, n (%)	Mean Sub-Score	Contribution to Mean Total (%)	Survivors, Median (IQR)	Non-Survivors, Median (IQR)
Respiratory	15 (65.2)	1.22	22.0	1 (0 to 2)	1 (1 to 3)
Cardiovascular	3 (13.0)	0.13	2.4	0 (0 to 0)	0 (0 to 0)
Hepatic	21 (91.3)	2.04	37.0	2 (1 to 2)	2 (2 to 4)
Renal	11 (47.8)	1.43	26.0	0 (0 to 3)	2 (0 to 4)
Coagulation	5 (21.7)	0.30	5.5	0 (0 to 1)	0 (0 to 0)
Neurological	8 (34.8)	0.39	7.1	0 (0 to 0)	1 (1 to 1)

IQR, interquartile range. Contribution to the mean total score was calculated as mean component score divided by mean total score, multiplied by 100. Median sub-scores are given separately for survivors and non-survivors as a description of the cohort; no statistical comparison between the outcome groups was performed. A sub-score above zero indicates that the measured variable crossed the prespecified threshold of the applied scoring scheme; it should not be interpreted as an independently validated diagnosis of organ dysfunction.

## Data Availability

The individual-level dataset supporting the findings of this study is available from the corresponding author upon reasonable request.

## Supplementary Materials

The following supporting information can be downloaded at: https://www.mdpi.com/article/10.3390/life16091575/s1, Table S1: Sensitivity and specificity of the applied total score at each attainable cut-off.

## Abbreviations

The following abbreviations are used in this manuscript:

APPLE	Acute Patient Physiologic and Laboratory Evaluation
FiO_2_	fraction of inspired oxygen
IQR	interquartile range
IRIS	International Renal Interest Society
MAP	mean arterial pressure
mGCS	modified Glasgow Coma Scale
PaO_2_	arterial oxygen tension
SAP	systolic arterial pressure
SIRS	systemic inflammatory response syndrome
SOFA	Sequential Organ Failure Assessment
SpO_2_	peripheral oxygen saturation
